# Epigenetic Modifiers Are Necessary but Not Sufficient for Reprogramming Non-Myelinating Cells into Myelin Gene-Expressing Cells

**DOI:** 10.1371/journal.pone.0013023

**Published:** 2010-09-27

**Authors:** Jia Liu, Juan Sandoval, Sung Tae Doh, Li Cai, Gerardo López-Rodas, Patrizia Casaccia

**Affiliations:** 1 Department of Neuroscience, Mount Sinai School of Medicine, New York, New York, United States of America; 2 Department of Biochemistry and Molecular Biology, University of Valencia, Valencia, Spain; 3 Department of Biomedical Engineering, Rutgers University, Piscataway, New Jersey, United States of America; University of Washington, United States of America

## Abstract

**Background:**

Modifications on specific histone residues and DNA methylation play an essential role in lineage choice and cellular reprogramming. We have previously shown that histone modifications or combinatorial codes of transcription factors (TFs) are critical for the differentiation of multipotential progenitors into myelinating oligodendrocytes. In this study we asked whether combining global manipulation of DNA methylation and histone acetylation together with the expression of oligodendrocyte- specific TFs, was sufficient to switch the identity of fibroblasts into myelin gene-expressing cells.

**Methodology/Principal Findings:**

Transfection of six oligodendrocyte-specific TFs (*Olig1*, *Olig2*, *Sox10*, *Mash1*, *E47* and *Nkx2.2*) into NIH3T3 fibroblasts was capable of inducing expression of myelin gene promoter-driven reporters, but did not activate endogenous myelin gene expression. These results suggested the existence of a transcriptionally incompetent chromatin conformation in NIH3T3 fibroblasts. Using chromatin immunoprecipitation (ChIP) analysis, we compared the histone code on the conserved regions of myelin genes (i.e. *Mbp* and *Mag*) in differentiating oligodendrocyte progenitors and NIH3T3 fibroblasts. Chromatin at myelin gene loci was characterized by the presence of repressive histone modifications (me3K9H3 and me3K27H3) in NIH3T3 fibroblasts and active histone marks (me3K4H3 and AcH3) in oligodendrocyte lineage cells. To induce a transcriptionally competent chromatin signature, NIH3T3 fibroblasts were treated with 5-azadeoxy-citidine (5-AzaC) to decrease DNA methylation, and trichostatin A (TSA) or sirtinol, to favor histone acetylation. Treatment with 5-AzaC/TSA but not sirtinol, resulted in the detection of endogenous myelin gene transcripts in fibroblasts, although not to the levels detected in myelinating cells. Transfection of oligodendrocyte-specific TFs after 5-AzaC/TSA treatment did not further increase myelin gene expression, nor did it reprogram the transcriptional network of NIH3T3 fibroblasts into that of oligodendrocytes.

**Conclusions/Significance:**

These results suggest that reprogramming of fibroblasts into myelin gene-expressing cells not only requires transcriptional activation, but also chromatin manipulations that go beyond histone acetylation and DNA methylation.

## Introduction

Epigenetic gene regulation plays an important role in the control of cell proliferation, specification and differentiation [1], [2]. Recent studies have shown that treatment with inhibitors of histone deacetylases (Hdacs), histone methyltransferases and/or DNA methyltransferases favor the efficiency of reprogramming somatic fibroblasts into induced pluripotent stem (iPS) cells [3], [4], [5] or neural-like cells [6], [7]. However, it is unclear if a non-myelinating cell (e.g. NIH3T3 fibroblasts) can be directly reprogrammed into a myelin-gene expressing cell by global epigenetic manipulation.

The specific modifications on individual histone amino acid residues determine the accessibility of chromatin, thereby affecting gene expression [1]. The balance of active and repressive histone modifications results in the establishment of a “histone code”, which together with DNA methylation, determines the specific lineage choice and defines the differentiation stage of a cell.

Oligodendrocytes are the myelin forming cells in the central nervous system. They derive from multipotential progenitors that develop into mature cells through a complex interplay between cell specific transcription factors (TFs) and epigenetic modulators, including microRNAs, histone modifiers and DNA methylation [8], [9]. Several studies have reported a critical role of histone modifications during the differentiation of oligodendrocytes in development and repair [10], [11], [12], [13]. Histone deacetylation is one of the first characterized modifications and is typically associated with gene repression [1], [2] We have previously shown that histone deacetylation, mediated by Hdacs decreases the expression of transcriptional regulators, such as *Id4* and *Tcf7l2*, effectively relieving the inhibition on myelin gene expression during oligodendrocyte development [10], [11], [12]. In animal models of demyelinating diseases pharmacological inhibition of Hdac or inefficient recruitment to the promoters of these genes, due to aging, resulted in high levels transcriptional inhibitors and inefficient remyelination [11]. Microinjections of antisense *hdac1* oligonucleotides successfully prevented differentiation of oligodendrocytes in zebrafish [14] and targeted deletion of *Hdac1* and *Hdac2* in mice did not allow proper formation of myelin during early postnatal life [15]. In addition, Hdac11 was previously reported to facilitate *Myelin basic protein* (*Mbp*) and *Proteolipid Protein (Plp)* gene expression, although the molecular mechanisms remain not completely understood [16].

Several elegant studies have addressed the role of lineage-specific transcription factors in oligodendrocyte lineage progression (reviewed in [17]) and supported their identification with genetic animal models. However, transfection of combinations of oligodendrocyte-specific TFs in unrelated cell lineages (i.e. fibroblasts) was sufficient to activate the expression of luciferase reporters driven by myelin gene promoters [18], but unable to activate endogenous myelin gene expression. We reasoned that these differences must be consequent to specific chromatin landscapes characteristic of each cell type and this was the premise for the current study.

In this study, we first defined the histone code on conserved regions of myelin genes in progenitors and differentiated primary oligodendrocytes and then compared the results with those obtained in myelinating cell lines (i.e. Oli*neu* cells) or in fibroblast cells (NIH3T3). We then postulated that global manipulations of the histone code using chromatin modifying agents could change the cell-specific chromatin conformation of fibroblasts and render it more prone to activation by transfected oligodendrocyte-specific TFs. To test this hypothesis, we treated fibroblasts with the DNA demethylating agent 5-AzaC and either the Hdac inhibitor TSA or the sirtuin inhibitor sirtinol and then transfected fibroblasts with six oligodendrocyte-specific TFs (*Olig1*, *Olig2*, *Mash1*, *Sox10*, *Nkx2.2* and *E47*). Although treatment of 5-AzaC and TSA enhanced transcription of endogenous myelin genes in fibroblasts, the additional over-expression of cell specific transcriptional regulators did not further enhance myelin gene transcript levels. These findings suggest that global epigenetic modifications are necessary, but not sufficient to trans-differentiate fibroblasts into oligodendrocytes, and underscore the importance of chromatin compaction and the associated repression of inhibitory events for the differentiation of myelinating cells.

## Results

### Oligodendrocyte-specific transcription factors fail to activate endogenous myelin gene expression in fibroblasts

**Figure 1:**
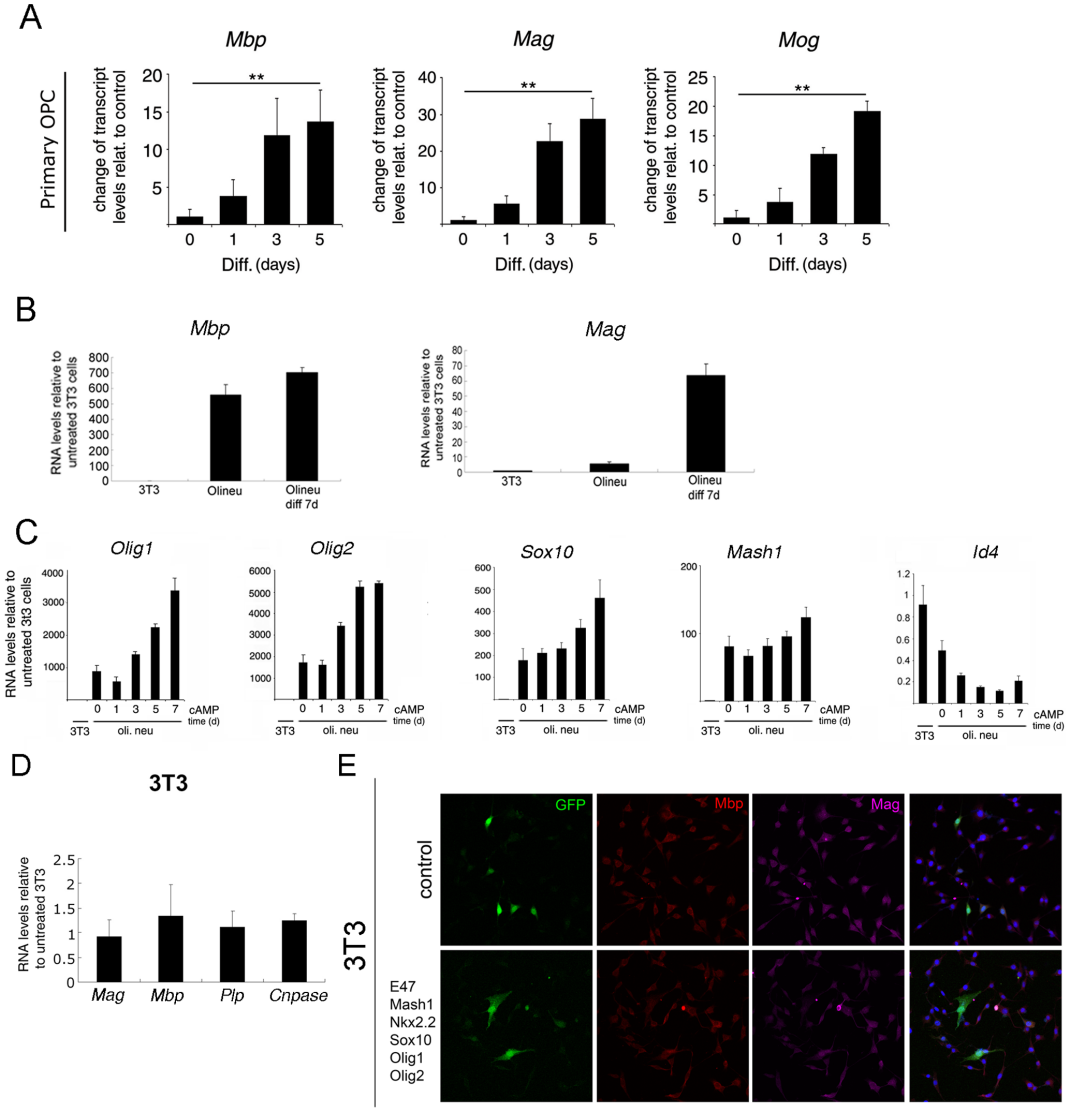
Transfection of oligodendrocyte-specific transcription factors is not sufficient to express endogenous myelin genes in fibroblasts. Temporal pattern of regulation of myelin gene expression in rat primary oligodendrocyte progenitors during differentiation (A). Transcript levels of *Mbp*, *Mag* and *Mog* were analyzed by quantitative RT-PCR in rat primary progenitors and in differentiated cells at the indicated time points (1, 3 and 5 days). Transcript levels of myelin genes (B) and oligodendrocyte-specific transcription factors (C) were analyzed by qRT-PCR in non-myelinating (NIH3T3) and in myelinating (Oli*neu*) cell lines differentiated with cAMP for multiple time points (DIV 0,1,3,5,7). The effect of transfecting combinations of positive transcription factors (*E47*, *Mash1*, *Nkx2.2*, *Sox10*, *Olig1* and *Olig2*) was assessed by detecting myelin gene transcripts using qRT-PCR (D) and myelin proteins, using immunocytochemistry (E). Values were normalized by the levels of *Gapdh* and shown as relative to the levels detected in untreated sample. The bar graphs represent the average values and error bars represent standard deviations (n = 3; * p<0.05; ** p<0.01; *** p<0.001).

In this study, we first analyzed the levels of *Mbp*, *Myelin associated glycoprotein* (*Mag*) and *Myelin oligodendrocyte glycoprotein* (*Mog*) transcripts as a measure of the differentiation of primary oligodendrocyte progenitor cells (OPCs) into mature oligodendrocytes (Fig. 1A). Mbp is one of the most abundant proteins in myelin of the central nervous system (CNS) and is required for the formation of a compact structure [19]. Mag is the protein that is in contact with the axon, while Mog is one of the latest genes to be transcribed during differentiation and encodes for a protein that is required for myelin maintenance [19]. As expected, analysis of RNA samples by quantitative RT-PCR showed increased myelin gene transcripts as OPCs were kept for one to five days in differentiation conditions *in vitro*. This pattern was then compared with the one obtained in the immortalized oligodendrocyte progenitor cell line Oli*neu* [20] after differentiation and with the endogenous levels of these myelin gene transcripts in the fibroblast cell line, NIH3T3 (Fig. 1B). Myelin genes were not detected in fibroblasts, but were expressed in Oli*neu*, although the temporal increase was not as robust as the one detected in primary cells (Figure 1B). As expected, fibroblasts expressed very low levels of transcriptional activators (*Olig1*, *Olig2*, *Sox10* and *Mash1*), in the order of hundreds or thousands fold lower than Oli*neu* and higher levels of transcriptional inhibitors (*Id4*) (Figure 1C). A potential explanation for the lack of myelin expression in fibroblasts therefore could be the absence of a threshold level of transcriptional activators to overcome the negative effect of the transcriptional inhibitors. Indeed, we had previously shown that overexpression of oligodendrocyte-specific transcription factors (*Olig1*, *Olig2*, *Sox10*, *E47*, *Mash1*, and *Nkx2.2*) in NIH3T3 cells was sufficient to transiently activate exogenous luciferase reporters driven by the *Mbp* promoters [18]. We therefore attempted to artificially increase the levels of the oligodendrocyte-specific TFs by transfecting them into NIH3T3. However, the over-expressing cells failed to show increased endogenous myelin gene expression (Figure 1D and 1E). Thus, the transfection of combination of oligodendrocyte-specific transcriptional activators was sufficient to drive the expression of exogenously added *Mbp* promoter-driven reporters, but was not sufficient to drive endogenous expression of myelin genes. The discordance of these two results suggested that the accessibility of TFs to endogenous myelin genes in fibroblasts might be compromised, due to a non-permissive chromatin environment that is present in NIH3T3 cells.

### Specific patterns of histone code in myelinating and non-myelinating cells

**Figure 2:**
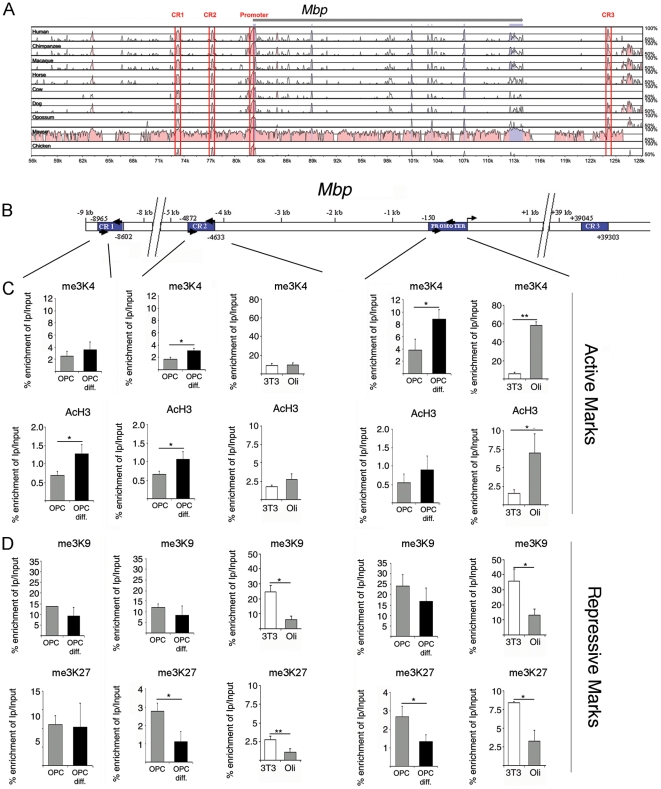
Comparison of the chromatin regions at the *Mbp* locus between oligodendrocyte progenitors and fibroblasts. Comparative sequence analysis of conserved (pink) and transcribed (blue) regions of the *Mbp* promoter in different animal species (A). Schematic representation of conserved regulatory regions (blue boxes) of the *Mbp* promoter (B). Comparative ChIP analysis of samples isolated from NIH3T3, Oli*neu* cells (Oli), proliferating (OPC) and differentiating (OPC diff) oligodendrocyte progenitors (C). Histone modifications in the different *Mbp* promoter regions were assessed by immunoprecipitating chromatin with antibodies specific for the activating histone marks (me3K4H3 and AcH3) (C) or for repressive histone modifications (me3K9H3 and me3K27H3) (D). The *Mbp* locus was characterized by permissive histone marks in oligodendrocyte progenitors and non-permissive marks in fibroblasts. ChIP was performed on five separate cultures. Results are expressed as mean ± standard deviation (SD) and statistically analyzed using two tailed Student's *t* tests (*p<0.05, **p<0.01).

It is well accepted that the accessibility of chromatin to transcription factors is dependent on post-translational modification of histones that determine the conformational state of chromatin. For this reason, we sought to define the “histone code” of myelin gene loci in NIH3T3 fibroblasts and compared it with that detected in myelinating cell lines (i.e. Oli*neu* cells) and in undifferentiated OPCs and mature oligodendrocytes. We focused our analysis on highly conserved regions in the myelin genes (*Mbp* and *Mag*) with the assumption that these conserved elements play important regulatory functions. To identify highly conserved regions in *Mbp*, we used the NCSRS software [21] and performed multiple sequence alignments of homologous sequences using the rat genome as a baseline. These alignments identified one conserved region in the proximal promoter of *Mbp* (close to the transcription start site), two conserved regions further 5′ upstream (CR1 and CR2) and one conserved region in the 3′ transcript region (CR3) (Figure 2A and 2B). The conserved modules identified within the *Mbp* promoter and upstream sequence contain the four regulatory regions, previously identified using transgenic approaches *in vivo* [22]. In particular, CR2, which coincides with the M3 element, corresponds to a conserved region that confers high levels of expression in oligodendrocytes in the CNS [22]. For this reason we focused our analysis on the characterization of the histone code at the CR2 and the proximal promoter region.

The histone code used by different cell types to allow or limit the accessibility of transcription factors to myelin gene loci, was then studied using chromatin immunoprecipitation (ChIP) with antibodies against histone marks associated with transcriptional activity (i.e. trimethylated lysine 4 on histone 3 (me3K4H3) and pan acetylated H3 (AcH3)) as well as those associated with transcriptional repression (i.e. trimethylated lysine 9 on histone 3 (me3K9H3) and trimethylated lysine 27 on histone 3 (me3K27H3)). Chromatin was isolated from progenitors and differentiated primary oligodendrocyte cultures and the experiment was repeated five times on freshly prepared samples. After immunoprecipitation with the antibodies, the conserved regions of the *Mbp* locus were analyzed.

The chromatin regions corresponding to the promoter and conserved upstream regulatory regions of the *Mbp* locus were characterized by the presence of both active (me3K4H3 and AcH3) and repressive (me3K9H3 and me3K27H3) marks in undifferentiated primary OPCS(Fig. 2C–D). The presence of both marks of activation (me3K4H3) and repression (me3K27H3) has also been defined as “bivalent mark” [23], [24] and has been reported to characterize a state of transcriptional competence. As OPCs differentiate into oligodendrocytes, there is a resolution of the bivalent marks at the CR2 and proximal promoter regions. The chromatin at these regions is characterized by decreased me3K27H3 and increased me3K4H3 and to a lower extent increased panAcH3 (Fig. 2C–D). This histone code is consistent with the expression of *Mbp* transcripts during this transition (Figure 1A). Therefore, the expression of *Mbp* during the differentiation of primary OPCs into oligodendrocytes is characterized by the deposition of active methylation marks on lysine 4 and removal of repressive methylation marks on lysine 27 of histone H3 in chromatin at the highly conserved regions of the *Mbp* locus.

We then asked whether the chromatin at the *Mbp* locus of the myelinating cell line Oli*neu* and of the non-myelinating line NIH3T3, displayed a similar distribution of active and repressive marks. While the chromatin isolated from Oli*neu* cells was characterized by the presence of bivalent marks, similar to primary OPCs, the most remarkable difference between the myelinating and non-myelinating lines was the prominence of a repressive histone code in NIH3T3 cells, characterized by me3K9H3 and me3K27H3 at the CR2 and promoter region of *Mbp* (Figure 2D). These data suggest that distinct cell types adopt precise histone codes to modulate the accessibility of TFs to the chromatin regions containing myelin genes.

**Figure 3:**
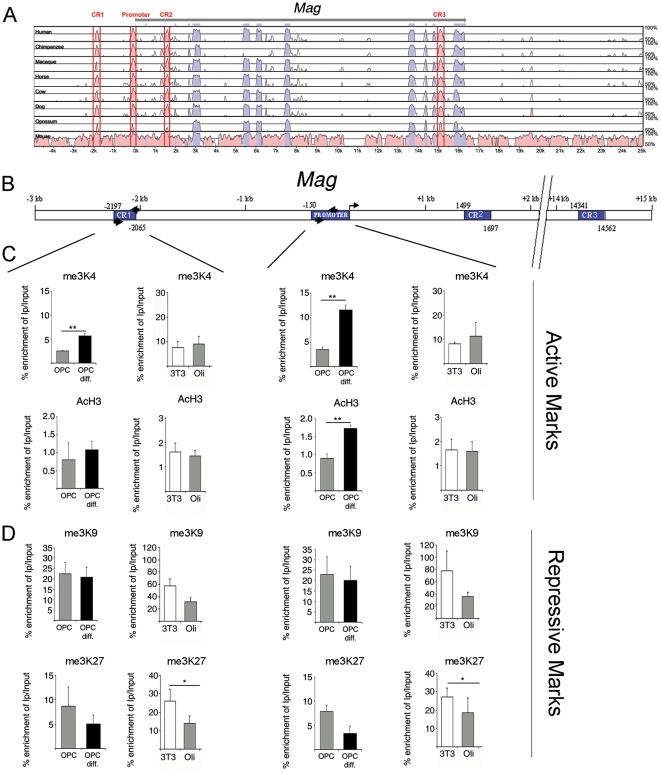
Comparison of the chromatin regions at the *Mag* gene between oligodendrocyte progenitors and fibroblasts. Comparative sequence analysis of conserved (pink) and transcribed regions (blue) of the *Mag* promoter in different animal species (A). B, Schematic representation of conserved and/or previously reported regulatory regions of the *Mag* promoter. Schematic representation of conserved regulatory regions (blue boxes) of the *Mag* promoter (B). Comparative ChIP analysis of samples isolated from NIH3T3, Oli*neu* cells (Oli), proliferating (OPC) and differentiating (OPC diff) oligodendrocyte progenitors (C). Histone modifications in the different *Mag* promoter regions were assessed by chromatin immunoprecipitation using antibodies specific for the active histone marks me3K4H3 and AcH3(C) and for the repressive histone modifications me3K9H3 and me3K27H3 (D). The chromatin regions of the *Mag* gene were characterized by permissive histone marks in oligodendrocyte progenitors and non-permissive marks in fibroblasts. ChIP was performed on five separate cultures. Results are expressed as mean ± standard deviation (SD) and statistically analyzed using two tailed Student's *t* tests. (*p<0.05, **p<0.01).

We then performed a similar sequence alignment for the *Mag* gene and identified one conserved region in the proximal promoter, one in the distal promoter (CR1) and two in the transcribed region (CR2 and CR3) (Figure 3A–B). The chromatin of undifferentiated OPCs at the CR1 and promoter regions of the *Mag* locus was in a transcriptionally competent state characterized by the bivalent mark (i.e. me3K4H3 and me3K27H3) (Figure 3C–D). As for *Mbp*, the differentiation of OPCs into oligodendrocytes was characterized by a resolution of the bivalent code also on the *Mag* gene with increased me3K4H3 and decreased me3K27H3 at the promoter and CR1 region. These modifications were specific to myelin genes, as the histone marks did not change in the promoter region of *β-actin* during OPCs differentiation (Figure S1).

Intriguingly, a comparison of the histone code of *Mag* in non-myelinating NIH3T3 and myelinating Oli*neu* cell lines, revealed that the greatest difference was at the level of repressive mark, with fibroblasts being characterized by the prominent presence of me3K27H3 and me3K9H3, compared to Oli*neu* cells (Figure 3C–D).

From these combined data we conclude that distinctive histone modifications at myelin gene loci modulate the accessibility of TFs to DNA in distinct cell types. Primary OPCs and myelinating cell lines are characterized by the presence of a transcriptionally competent and accessible state of chromatin. Differentiation into mature OL occurs when chromatin reorganization leads to a transcriptionally active state characterized by the presence of activating TFs. In non-myelinating cell lines, in contrast, the chromatin is characterized by a repressive histone code and this renders the myelin gene loci inaccessible to transcriptional activation.

### Treatment of fibroblasts with global epigenetic modifiers increases the expression of myelin genes in NIH3T3 Cells

**Figure 4:**
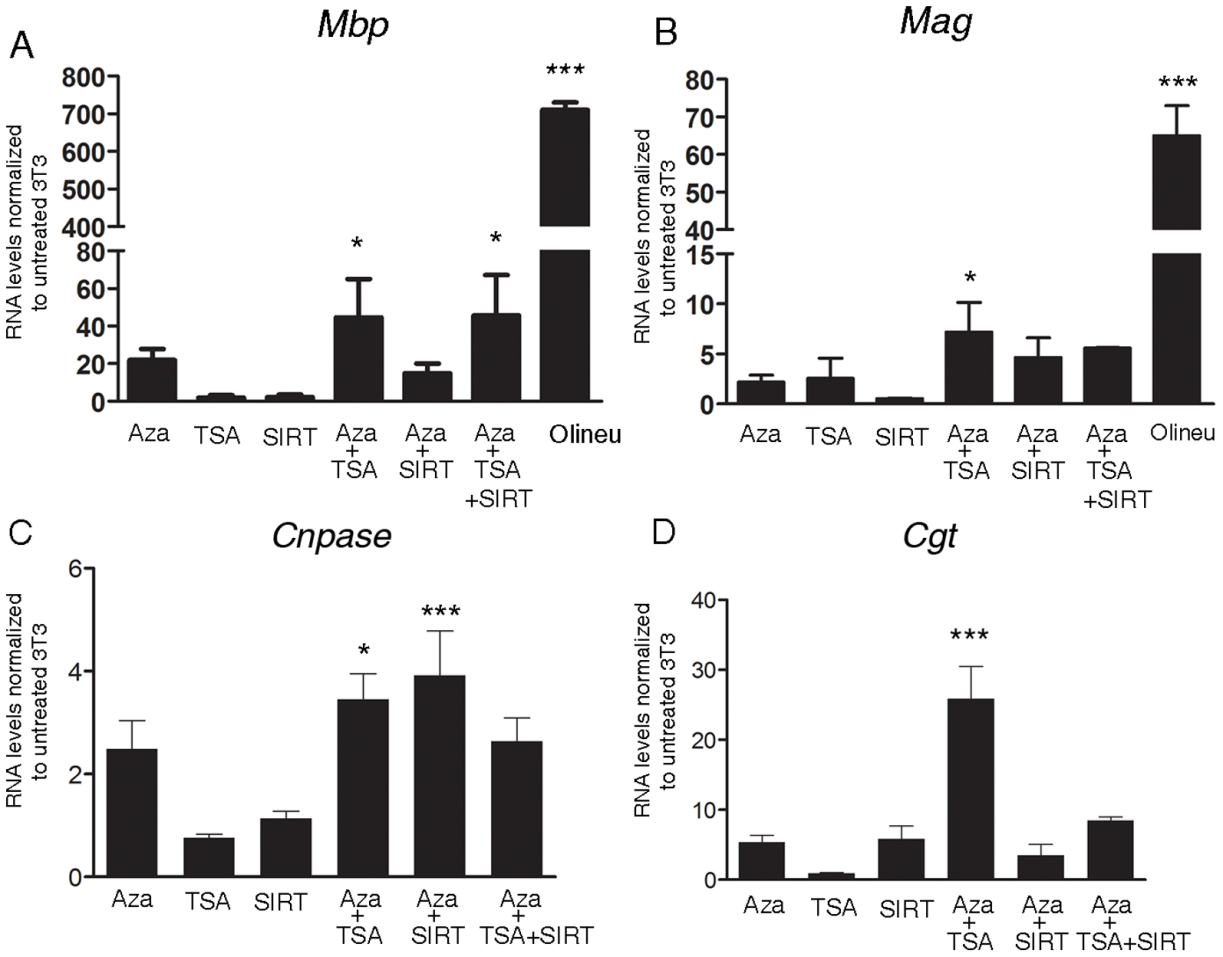
Endogenous myelin gene expression in fibroblasts can be elicited by global pharmacological epigenetic modulators. NIH3T3 cells were treated with the DNA demethylating agent, 5-azacytidine (5-AzaC), and/or with the histone deacetylases inhibitors trichostatin A (TSA) and sirtinol. The transcript levels of *Mbp* (A), *Mag* (B), *Cnpase* (C) and *Cgt* (D) were analyzed by qRT-PCR. Endogenous myelin genes were expressed in fibroblasts treated with 5-AzaC and TSA, but not in untreated cells, even though they did not reach the levels of myelinating cells (Oli*neu* cells in A and B). Also, treatment with 5-AzaC/sirtinol was less effective than 5-AzaC/TSA. Experiments were performed on three independent cultures. Results are expressed as mean ± standard error (SE) and statistically compared with the levels detected in untreated NIH3T3 cells using a one way ANOVA test followed by *post hoc* analysis. (*p<0.05, **p<0.01, ***p<0.001).

The detection of a repressive histone code at myelin gene loci in fibroblasts suggested the possible co-existence of additional mechanisms of epigenetic silencing, including DNA methylation [25], [26] and histone deacetylation [27], [28], and prompted the question of whether relieving such inhibition with global epigenetic modifiers, would lead to increased expression of myelin genes. For this reason, we treated NIH3T3 cells with the demethylating agent 5-AzaC, the class I and II Hdac inhibitor TSA and with the sirtuin inhibitor sirtinol either alone or in sequential combination (i.e. 5-AzaC for 3 days followed by TSA or sirtinol for 1 day), and then measured endogenous myelin gene expression. Although no single treatment was effective by itself, the combination of 5-AzaC followed by TSA was sufficient to induce the endogenous expression of *Mbp*, *Mag*, *Cnpase* and *Cgt* in fibroblasts, whereas treatment with 5-AzaC and sirtinol was able to elicit this effect only for *Cnpase* expression (Figure 4A–D).

**Figure 5:**
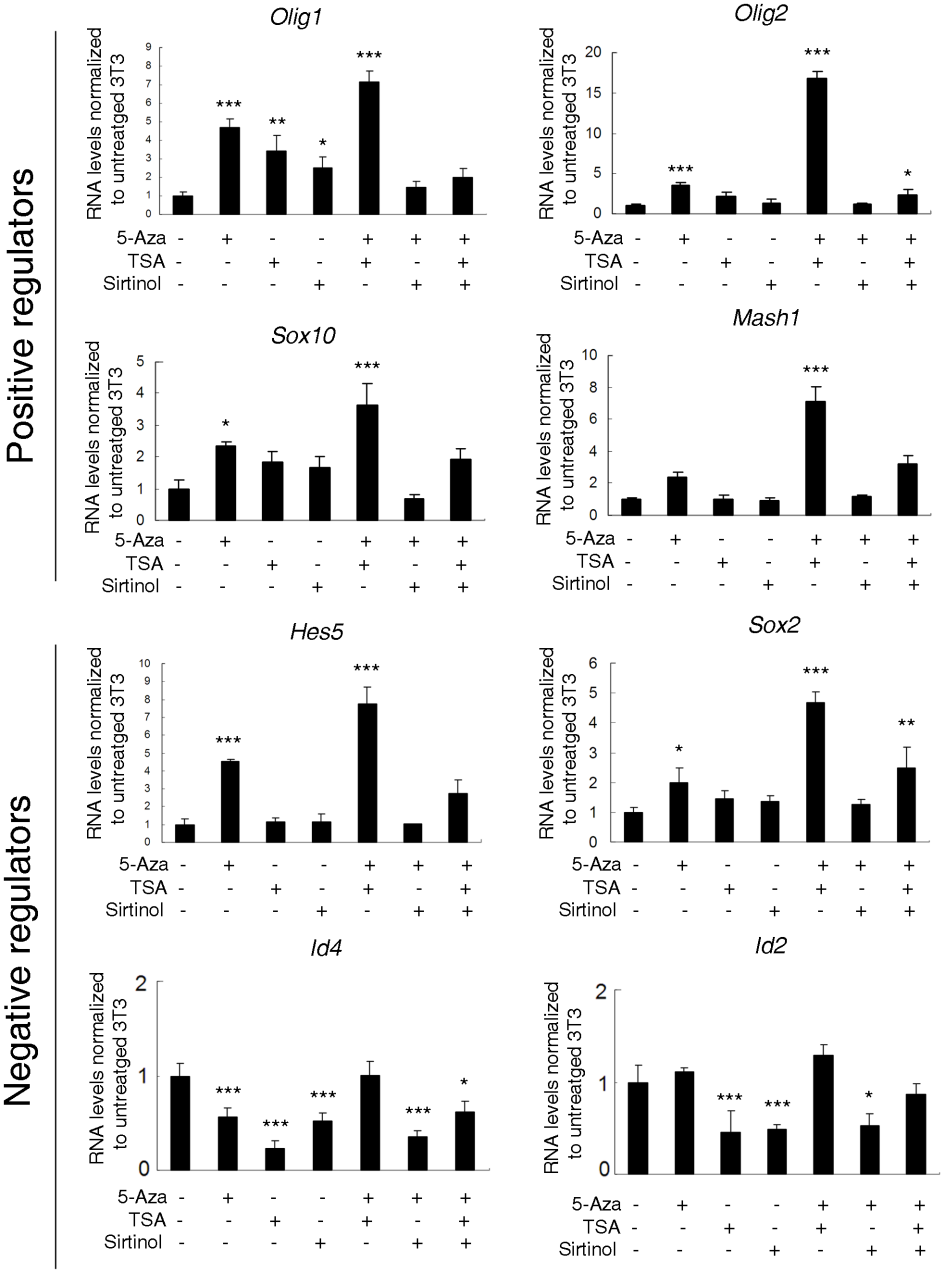
Combinational treatment with epigenetic modifiers induced endogenous oligodendrocyte-specific transcription factor expression in NIH3T3 cells. NIH3T3 cells were treated with the DNA demethylating agent 5-azacytidine (5-AzaC) and/or with the histone deacetylase inhibitor trichostatin A (TSA) and with the sirtuin inhibitor sirtinol alone or in combination. Positive (*Olig1*, *Olig2*, *Sox10* and *Mash1*) and negative (*Hes5*, *Sox2*, *Id4 and Id2*) transcriptional regulators of myelin genes were up-regulated in NIH3T3 cells treated with 5-AzaC and TSA. Experiments were performed in two independent cultures. Results are expressed as mean ± standard error (SE) and statistically compared with the levels detected in untreated 3T3 cells, using one way ANOVA followed by *post hoc* analysis(*p<0.05, **p<0.01, ***p<0.001).

In addition to activating myelin gene expression, the combination 5-AzaC and TSA also caused a statistically significant increase of the transcript levels of *Olig1* (p<0.001), *Olig2* (p<0.001), *Mash1* (p<0.005) and *Sox10* (p<0.01) and of the transcriptional inhibitor *Hes5* (p<0.001), *Sox2* (p<0.001) while *Id4* and *Id2* were not affected (Figure 5). Therefore the treatment of fibroblasts with broad epigenetic modifiers up-regulated several transcripts, although the overall pattern of gene expression did not match the precise pattern detected in myelinating cells (Figure 1). We have previously shown that myelin gene expression in myelinating cells requires the down-regulation of transcriptional inhibitors [11] and the up-regulation of transcriptional activators to levels that are several hundred folds higher than those measured in treated fibroblasts. We therefore reasoned that over-expression of TFs could be necessary to impact on the amount of myelin gene expression.

### Combination of epigenetic modifiers and expression of oligodendrocyte-specific transcription factors in fibroblasts did not further enhance myelin gene expression

**Figure 6:**
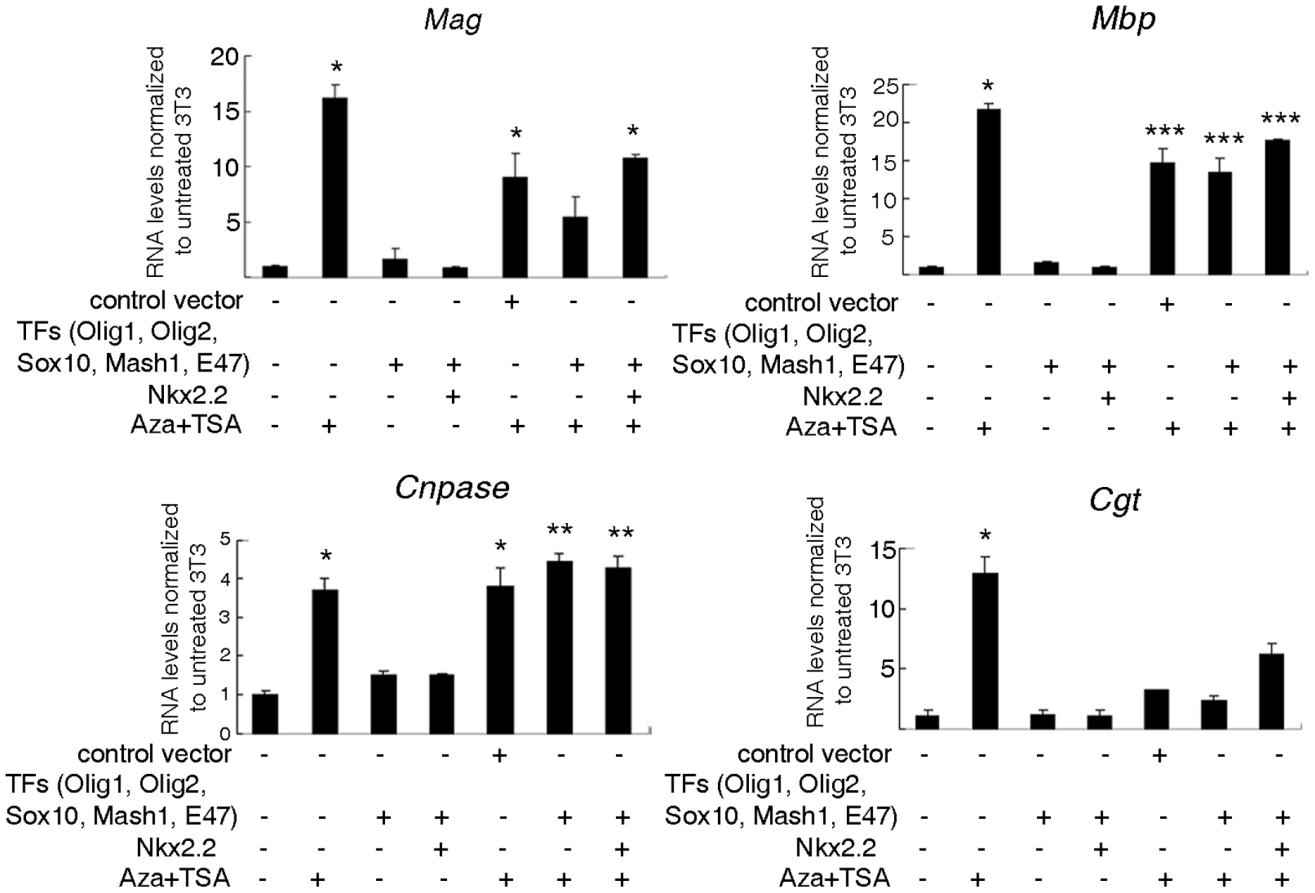
Oligodendrocyte-specific TFs after treatment with global modifiers did not further enhance myelin gene expression. NIH3T3 cells were treated with 5-AzaC for 3 days and TSA for 1 day and then transfected with oligodendrocyte-specific combinations of transcription factors (*Olig1*, *Olig2*, *Sox10*, *Mash1* , *E47* with or without *Nkx2.2*). RNA was harvested 48 hours later and processed for qRT-PCR. Experiments were performed in three independent cultures. Results are expressed as mean ± standard error (SE) and statistically compared with the levels detected in untreated NIH3T3 cells using one way ANOVA followed by *post hoc* analysis (*p<0.05, **p<0.01, ***p<0.001).

To test the hypothesis that both the levels of transcription factors and specific post-translational histone modifications are necessary to achieve high levels of myelin gene expression in fibroblasts, we combined the pharmacological manipulation of NIH3T3 cells with chromatin modifying agents with the over-expression of oligodendrocyte specific TFs. Fibroblasts were treated with 5-AzaC for 3 days followed by TSA treatment for 1 day in order to remove the repressive mark and favor the responsiveness to over-expression of *Olig1*, *Olig2*, *Sox10*, *Mash1*, *E47* and *Nkx2.2*. Two days later the endogenous levels of myelin gene transcripts were quantified. Considering the previously reported inhibitory role of Nkx2.2 on the activity of *Mbp* promoter [29], we also performed the experiment in the absence of Nkx2.2. The effect of the combined transfection and treatment on the expression of myelin gene transcripts in fibroblasts was statistically significant, but there was not an additive effect on the myelin gene transcript levels (Figure 6).

**Figure 7:**
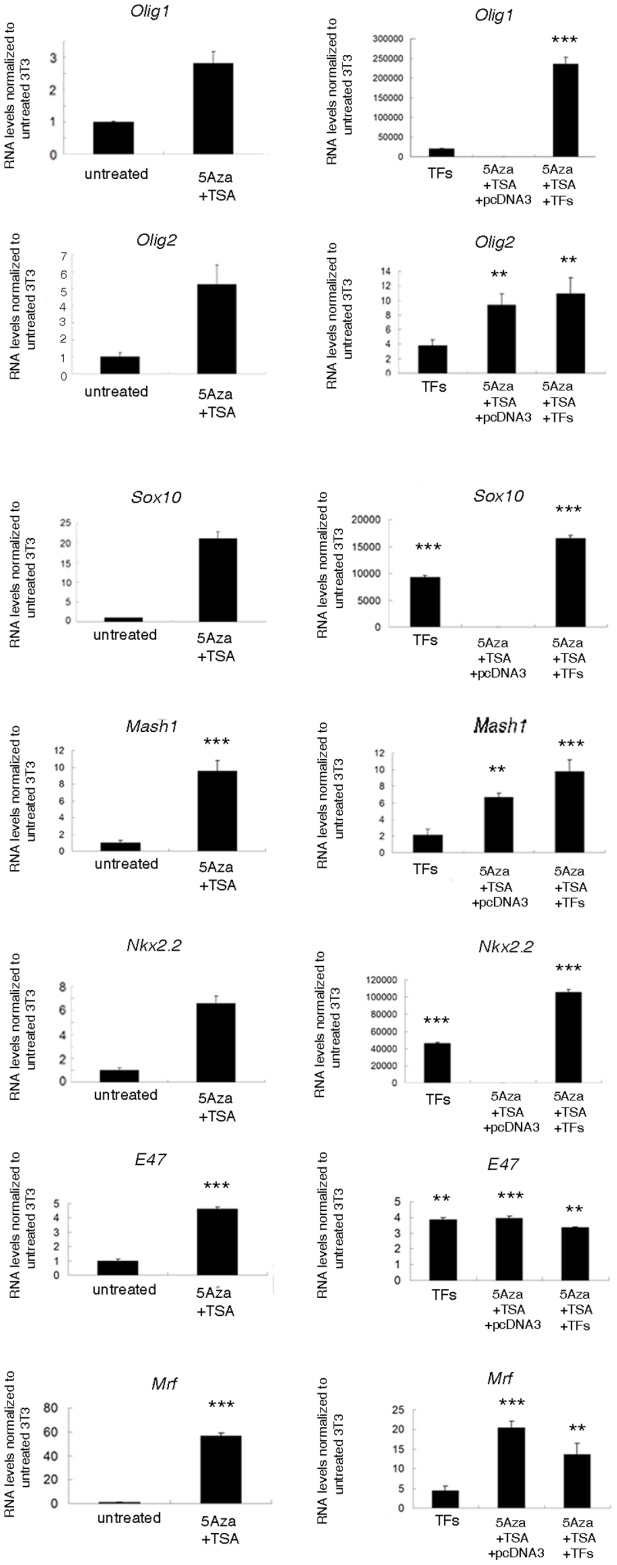
Expression of positive transcriptional regulators of myelin gene expression in manipulated NIH3T3. NIH3T3 cells were treated with 5-AzaC for 3 days and TSA for 1 day and then transfected with oligodendrocyte-specific combinations of transcription factors (*Olig1*, *Olig2*, *Sox10*, *Mash1*, *E47* and *Nkx2.2*). RNA was harvested 48 hours later and processed for qRT-PCR. Results are expressed as mean ± standard error (SE) and statistically compared with the levels detected in untreated NIH3T3 cells using two tailed Student's *t* test (*p<0.05, **p<0.01, ***p<0.001).

**Figure 8:**
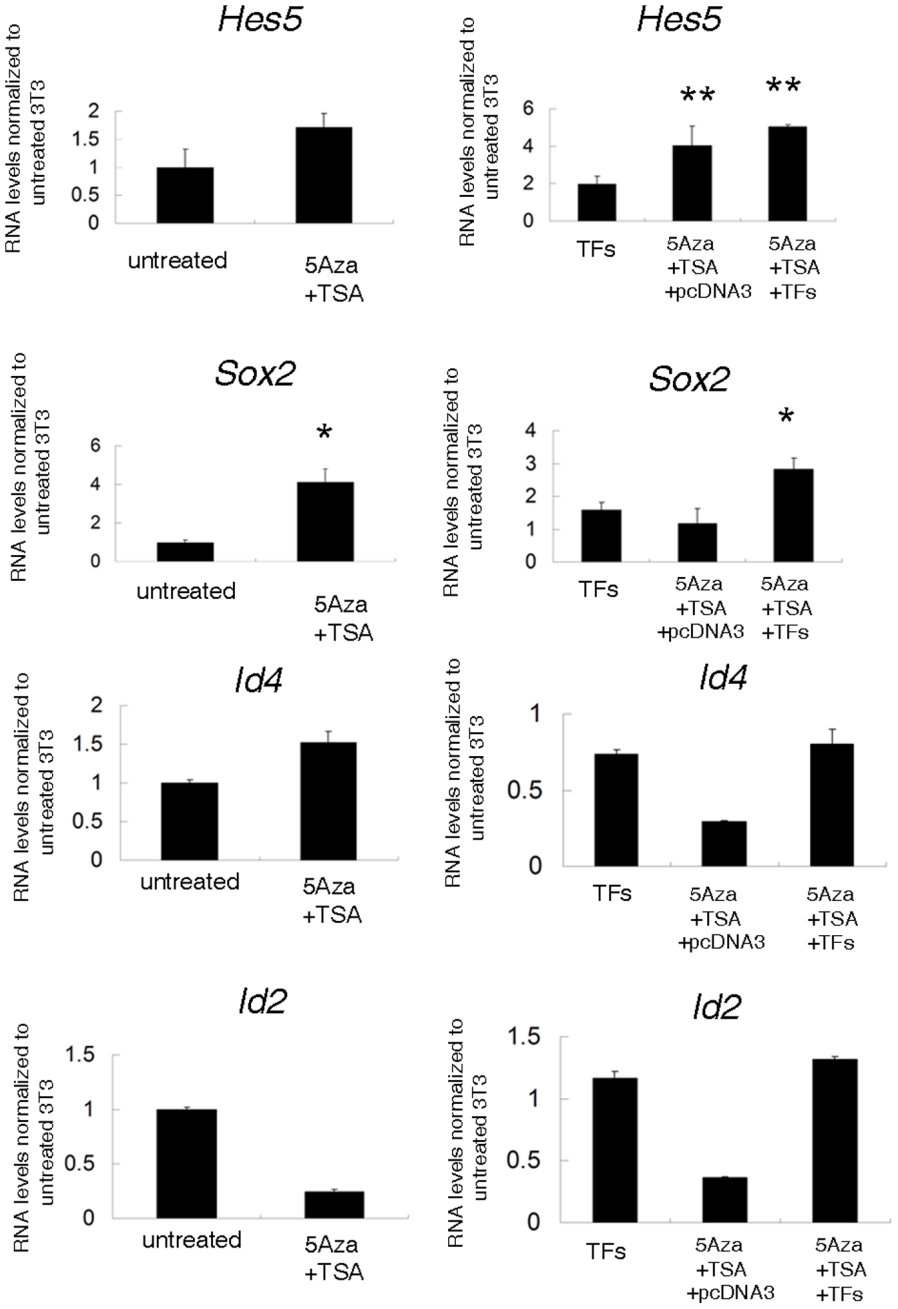
Expression of positive transcriptional regulators of myelin gene expression in manipulated NIH3T3. NIH3T3 cells were treated as described in figure 7. Experiments were performed in two independent cultures. Results are expressed as mean ± standard error (SE) and statistically compared with the levels detected in untreated NIH3T3 cells using two tailed Student's *t* tests (*p<0.05, **p<0.01, ***p<0.001).

Besides the obvious increase in the transcript levels of *Olig1*, *Olig2*, *Mash1*, *Sox10*, *Nkx2.2* and *E47* in the transfected cells we also detected the up-regulation of the endogenous transcriptional activator myelin gene regulatory factor (*Mrf*) (Figure 7), which is a recently identified TF for oligodendrocyte maturation and CNS myelination [30]. However, despite the high levels of these activators, the levels of myelin gene expression that were detected in fibroblasts did not reach the levels in myelinating cells. A potential explanation for these results was the simultaneous up-regulation of the transcriptional inhibitors. However, qRT-PCR measurement of the endogenous levels of *Hes5*, *Sox2*, *Id4* and *Id2* in 5-AzaC and TSA treated fibroblasts transfected with oligodendrocyte-specific TFs revealed a significant increase of *Hes5* and *Sox2*, but not *Id4* and *Id2* (Figure 8). We conclude that additional chromatin remodeling complexes, possibly coupled with the recruitment of co-activators are necessary for the massive induction of myelin gene expression detected in oligodendrocytes.

## Discussion

Cell identity is defined by the interplay of DNA methylation, histone modifications microRNAs and DNA binding proteins, which shapes the transcriptional profile of each cell type, by modulating the chromatin landscape. The modulation of these components has been exploited to reprogram fibroblasts into induced pluripotent stem cells (iPS) [31]. Recently, a combination of three defined factors (*Ascl1*, *Brn2* and *Myt1*) has been identified to be able to directly convert fibroblasts into induced neuronal (iN) cells, indicating that the cell identity switch could occur without going through an undifferentiated state [32].

Based on these studies, we have asked whether combinations of oligodendrocyte-specific TFs would be able to convert fibroblasts into myelin gene-expressing cells. However, these TFs were only able to activate myelin gene promoter-driven luciferase reporters, but were not sufficient to activate endogenous myelin expression or switch cell identity. A likely explanation for the inability of transfected TFs to generate myelin-expressing cells was the possible existence of a repressive histone code that would counteract the function of activating TFs. In the reprogramming of fibroblasts into iPS or neural cells, for instance, the efficiency of conversion is extremely low and this has been attributed to the need for a progressive accumulation of chromatin modifications, including changes in the histone code and in DNA marks that are associated with switching fates [33], [34]. Indeed, treatment of fibroblasts with compounds affecting histone and DNA modifications have been used to facilitate the conversion of fibroblasts into iPS cells [3], [4], [5]. Likewise, epigenetic modifications consequent to the use of pharmacological inhibitors of histone deacetylases, histone methyltransferases and/or DNA methyltransferases have been reported to favor the conversion of fibroblasts into “neural-like cells” [6], [7].

To begin understanding the histone code that defines the identity of an oligodendrocyte and characterizes the changes occurring during the differentiation process, we conducted ChIP assay with antibodies specific for activating and repressive histone modifications and compared the chromatin landscape at the *Mbp* and *Mag* loci of primary OPCs differentiating into oligodendrocytes. We then compared these results to those obtained from comparing the histone marks present at the same loci in myelinating and in fibroblast cell lines. The chromatin in primary progenitors and in myelinating cell lines was characterized by the presence of a transcriptionally competent histone code, composed of bivalent marks of activation (i.e. me3K4H3 and AcH3) and repression (me3K27H3). The coexistence of functionally opposite marks in immature progenitors and in myelinating cell lines was indicative of a “poised”, but inactive or weakly active transcriptional state at the myelin gene loci. Differentiation into myelinating oligodendrocytes coincided with the decrease of the repressive me3K27H3 mark and the increase of the active me3K4H3 marks in histones at the promoter regions of *Mbp* and *Mag*. It has been previously reported that decreased histone acetylation is part of the activating histone codes of myelin genes, although the precise mechanism has not yet been elucidated [16]. Our results did not detect significant deacetylation during the transition from progenitors to myelinating oligodendrocyte. We and others have previously reported that histone deacetylation is a critical event for the down-regulation of transcription factors defining the progenitor state [9], [17]. Combined with the results of this study, we suggest that the differentiation of progenitors into oligodendrocytes requires a series of sequential events and changes of histone marks at distinct loci. Our data suggest that deacetylation of critical lysine residues on histone H3 is an early event occurring at loci encoding for transcriptional inhibitors of myelin genes [10], [11], and is followed by decreased repressive me3K27H3 marks and increased activating me3K4H3 marks occurring at the loci encoding for myelin genes. Together, these histone changes are consistent with previous results reporting global histone deacetylation followed by histone methylation during the development of oligodendrocytes *in vivo* [12].

The distinctive feature of the chromatin changes detected at myelin gene loci in fibroblasts lines was the presence of a repressive histone code characterized by me3K27H3 and me3K9H3, which is consistent with the existence of a transcriptionally “incompetent” chromatin in fibroblasts. Because trimethylation of residue K9 on histone 3 has been previously reported to be associated with DNA methylation [25], it was likely that the repressive histone code in fibroblasts was associated with DNA methylation. For this reason, we decided to treat fibroblast lines with a pharmacological inhibitor of DNA methyltransferase and inhibitors of histone deacetylases to overcome the “transcriptionally incompetent” chromatin state. The detection of endogenous myelin gene transcripts in treated fibroblasts suggested that, even in the absence of oligodendrocyte-specific TFs, myelin genes could be expressed. These results, however, should be cautiously interpreted as potential reprogramming of fibroblasts into myelin-expressing cells, because the expression of myelin genes did not reach the levels reached in myelinating cells and was characterized by the co-existence of transcriptional inhibitors and activators. In order to facilitate the conversion of fibroblasts to oligodendrocytes, we then attempted to overcome the activity of the transcriptional inhibitors, by over-expressing transcriptional activators of myelin genes. This combinational treatment was able to up-regulate several transcriptional activators, including the recently identified oligodendrocyte differentiating factor *Mrf* [30], although the levels of myelin gene transcripts were still consistently lower than of the levels in myelinating cell lines.

The inability to achieve complete reprogramming of fibroblast into myelin-expressing cells even after manipulation of critical epigenome modifiers and transfection of oligodendrocyte-specific TFs could be due to several reasons. One possibility is that oligodendrocyte differentiation might require the critical expression of specific microRNAs [35], [36], [37] that were not included in our study. MicroRNAs have been involved in the removal of transcriptional inhibition. The over-expression of TFs and up-regulation of endogenous myelin regulators was not sufficient to induce myelin gene expression to the levels detected in oligodendrocytes due to the insufficient removal of transcriptional inhibition which is mediated by microRNAs. An alternative possibility is that we did not allow enough time to allow the fibroblasts to modify the “memory” of the previous cellular identity. This might be especially true, since our study was focused on a fibroblast cell line that has been passaged for a very long time, rather than on freshly isolated somatic fibroblasts. Nevertheless, the fact that myelin gene expression can be achieved, albeit at low levels, suggests the additional possibility that the additional involvement of SWI/SNF subunits and ATP-dependent chromatin remodelers might be required.

In conclusion, our data suggests a necessary but not sufficient role for global epigenetic modification in reprogramming fibroblast into myelinating cells. With further investigation of additional key events or factors necessary for this fate switch, this cellular system could provide an important tool for the design of new strategies aimed at cell replacement.

## Materials and Methods

### Cell culture and treatment

NIH3T3 fibroblast cells were grown in DMEM supplemented with 10% fetal bovine serum, 2 mM L-glutamine, 1 mM sodium pyruvate, 100 U/ml penicillin, and 100 g/ml streptomycin. The mouse oligodendrocyte precursor cell line Oli*neu* [20] were grown on poly-ornithine-coated culture dishes. The immature Oli*neu* cells were maintained in growth medium consisting of DMEM supplemented with 2 mM L-glutamine, 1 mM sodium pyruvate, 10 ng/ml biotin, 100 µg/ml apotransferrin, 100 µM putrescine, 20 nM progesterone, 30 nM sodium selenite, 5 µg/ml insulin, 1% horse serum, 100 U/ml penicillin, and 100 µg/ml streptomycin. Differentiation was induced by switching the cells to a serum-free medium containing 1 mM dibutyril-cAMP (Calbiochem), as described previously [20]. During the experiments where NIH3T3 cells were exposed to epigenetic modifying agents, cells were treated with 5µM 5-Aza-2′-deoxycytidine (Sigma), 10ng/ml trichostatin A (Sigma), and 20µM sirtinol (Sigma) respectively. NIH3T3 cells were transfected with HA-tagged *Olig1*, Flagged-tagged *Olig2* constructs inserted into pcDNA3.1, c-MYC-tagged *Nkx2.2* construct inserted into pcDNA3.1B, *Sox10*, *Mash1*, and *E47* full-length coding sequences that were inserted into pcDNA3.1 as previously described [18] using Lipofectamine 2000 (Invitrogen) according to the manufacturer's protocol.

Oligodendrocyte progenitors were isolated from the cortex of postnatal day 1 rats and grown for one week as mixed glial cultures as described previously [38]. Differential shaking followed by magnetic beads immunoselection was used to isolate progenitors. Briefly, cells were incubated with the A2B5 antibody, and purified using magnetic beads (Miltenyi Biotec, Auburn, CA) and then plated at a density of 2×10^5^ cells/ml on poly-D-lysine coated 10 cm plates. Progenitors were maintained proliferating by the addition of bFGF and PDGF (10 ng/ml) to the culture medium (DMEM, 100 µg/ml albumin, 100 µg/ml apo-transferrin, 16 µg/ml putrescine, 0.06 ng/ml progesterone, 40 ng/ml selenium, 5 µg/ml insulin, 1 mM sodium pyruvate, 2 mM l-glutamine, 100 units/ml penicillin, 100 µg/ml streptomycin). Differentiation was initiated by placing the cells in mitogen-free chemically defined cultured medium, whose composition is described above. For differentiating progenitors the treatment was performed in proliferating conditions and during 5 days of differentiation.

### Alignment of genomic sequences

Sequences and annotation used in the alignments were retrieved using NCSRS software [21]. This web based program returns the sequence of the upstream non-coding region, gene, and downstream non-coding region along with the associated annotation for any gene (References: NCSRS - PMID: 17362514 LAGAN - PMID: 12654723). It can also retrieve the corresponding sequences and annotations for the set of homologs of that gene. Select sequences and annotations are aligned with LAGAN using the translated anchoring option.

Non-coding regions that are conserved over multiple species are predicted to possess regulatory function. If these regions are within 1kb of a transcription start site then they are designated as promoters. Alignments show exon regions in blue (Figure 2A and 3A). Conserved regions have a sequence similarity of a minimum of 75% with the corresponding rat sequence for at least 100 base pairs and are shown in pink (Figure 2A and 3A). The limits of the conserved regions are taken to be as inclusive as possible without losing resolution. The limits of the conserved regions for many of the alignments tend to fall within those of the human / rat alignment. Therefore as a general rule the limits of the human / rat alignment are used to define the limits of the conserved regions.

### Quantitative RT-PCR

Oli*neu*, NIH3T3 or rat primary cells were homogenized in Trizol Reagent and RNA was isolated following manufacturer's instruction and cleaned using RNeasy Mini kit (Qiagen, Hilden, Germany). 2µg of total RNA was used in 20 µl of reverse transcription (RT) reaction, using SuperScript RT-PCR kit (Invitrogen). Quantitative real time PCR was performed using SYBR green PCR master mix (Applied Biosystems, CA) in 7900HT Sequence Detection PCR System (Applied Biosystems, CA). Melting curve of each sample was measured to ensure the specificity of the products. Data were normalized to the internal control *Gapdh* and analyzed using Pfaffl ΔΔCt method [39]. The primers used for amplification are listed in Table 1 for murine transcripts and Table 2 for rat transcripts.

**Table 1:** Primers used in quantitative PCR for amplification of mouse gene transcript.

Gene	Forward	Reverse
*Cgt*	TGGTTGACATACTGGATCACTATACT	CGATCACAAATCCACACATATCATT
*Cnpase*	TGGTGTCCGCTGATGCTTAC	CCGCTCGTGGTTGGTATCAT
*E47*	CACTGACCACGAGCTTCACC	AGGAGTCGGGAGGTCTCTGT
*Gapdh*	ACCCAGAAGACTGTGGATGG	CACATTGGGGGTAGGAACAC
*Hes5*	AGCAGCATAGAGCAGCTGAA	TAGTCCTGGTGCAGGCTCTT
*Id2*	GGACATCAGCATCCTGTCCT	CTCCTGGTGAAATGGCTGAT
*Id4*	CTGTCACCCTGCTTGTTCAC	GAGACTCACCCTGCTTTGCT
*Mash1*	GACGCTCTTGCTCCAGGAAA	CGAGAAGAGTGACTGGTGTCTGA
*Mbp*	ACACACGACAACTACCCATTATGG	AGAAATGGACTACTGGGTTTTCATCT
*Mag*	GGTGTTGAGGGAGGCAGTTG	CGTTCTCTGCTAGGCAAGCA
*Mrf*	TGGCAACTTCACCTACCACA	TGGCAACTTCACCTACCACA
*Nkx2.2*	ACACAGGTCAAGATCTGGTTCCA	GCGTCACCTCCATACCTTTCTC
*Olig1*	CGACGCCAAAGAGGAACAG	GCCAAGTTCAGGTCCTGCAT
*Olig2*	CCGAAGCAATGGGAGCAT	GGAGTGTTCAGCCAAAGAGTCA
*Plp*	CCCACCCCTATCCGCTAGTT	CAGGAAAAAAAGCACCATTGTG
*Sox2*	AACTTTTGTCCGAGACCGAGAA	CCTCCGGGAAGCGTGTACT
*Sox10*	GGAGATCAGCCACGAGGTAATG	GTTGGGTGGCAGGTATTGGT

**Table 2:** Primers used in quantitative PCR for amplification of transcripts in rat primary cultures.

Gene	Forward	Reverse
*18s rRNA*	AGTCCCTGCCCTTTGTACACA	GATCCGAGGGCCTCACTAAAC
*Mag*	GCTGGGAGGGAAATACTATTTCC	GACGCTGTGCTCTGAGAAGGT
*Mbp*	AAATCGGCTCACAAGGGATTC	CTCCCAGCTTAAAGATTTTGGAAA
*Mog*	GACGCACAGAGCTTCTGAAAGA	GAGAACCTCACGTTCTGGATCCT

### Chromatin immunoprecipitation

Chromatin was isolated from OPCs and from cells maintained in differentiation medium for 5 days. In brief, 4×10^6^ OPCs were trypsinized and cross-linked with 1% formaldehyde in suspension for 10 minutes and stopped with 0.125M glycine for 5 minutes. Chromatin was isolated with SDS lysis buffer and fragmented by sonication (6 cycles of 20 seconds at power level 6 in a Branson Sonifier 450). The size of the DNA in the sheared chromatin fragments was tested prior to precipitation, by performing agarose-gel electrophoresis to ensure that the majority of fragment size was 300–500bp. Immunoprecipitation was performed with 2.4 µg of anti-me3K4H3 (Abcam, ab8580), anti-me3K9H3 (Abcam, ab8898), anti-me3K27H3 (Upstate Biotechnology, 07-499) and anti-AcH3 (Abcam, ab8580) antibodies previously incubated with Protein A-coated paramagnetics beads (Invitrogen). The immunoprecipitated chromatin was reversed cross-linked, digested with proteinase K and DNA was extracted by phenol-chloroform. Immunoprecipitated samples were dissolved in 200 µL of TE and 5 µL were subjected to quantitative PCR amplification using the primers listed in Tables 3 and 4.

**Table 3:** Primers used for ChIP of mouse chromatin samples.

Gene	Region (nt)	Forward	Reverse
*Mag*	*Promoter*	GCCTGGAGCTTTCAGAAAGATGA	CTGGGACTGGGCAGCTTGAT
*Mag*	*CR1*	CCTATTCACCCTCAAAGTCCA	TTTCCCAGTCTGAGACAATGG
*Mbp*	*Promoter*	TTCAAGACCCCAGGAAGAAA	TTCTTTGGGTCTGCTGTGTG
*Mbp*	*CR2*	GTGGGTGGGTTGACAAGATT	GAGGGCACAGGAAACAAAAA

**Table 4:** Primers used for ChIP of rat chromatin samples.

Gene	Region (nt)	Forward	Reverse
*Mag*	*Promoter*	CTCCTCCCCTTCCTCCATTAA	GACAACAGGTTCCACCTTTCAAC
*Mag*	*CR1*	CGGGCTTCATGATGTCAGACT	CCCACTTTCAATGAACCATTGTC
*Mbp*	*Promoter*	TTCAAGACCCCAGGAAGAAA	TTCTTTGGGTCTGCTGTGTG
*Mbp*	*CR1*	GCAGCCACATGCCTCTCATA	GGAACAAAAAGGCCCATGGT
*Mbp*	*CR2*	GGCCTCTCTGTATCTCACAAATAACTG	CAAAACACAAGCACATCTGAATACTAAT
*betaactin*	*promoter*	CGCTGTGGCGTCCTATAAAA	CGAGGTAGTGGCCAAGGTAA

The DNA recovered from chromatin which was not immunoprecipitated (input) was used as a control for loading. Chromatin that was immunoprecipitated with protein A-agarose in the absence of primary antibody (no Ab) was used as a negative control.

### Statistical Methods

Results are expressed as mean ± standard deviation (SD) and statistically analyzed using two tailed Student's *t* tests or one way ANOVA followed by Dunnett's test. A value of *p*<0.05 was considered statistically significant. *p<0.05, **p<0.01, ***p<0.001.

## Supporting Information

Figure S1 The pattern of histone code on the βactin gene was similar during oligodendrocyte progenitor differentiation. The pattern of histone modifications in beta-actin proximal promoters was analyzed in proliferating (OPC) and differentiating (OPC diff) rat primary oligodendrocyte cultures. Note that the histone code of this constitutively expressed gene was similar at the two stages of differentiation.
(0.31 MB TIF)
