# Supplementary figures and images for: Epigenetic Modifiers Are Necessary but Not Sufficient for Reprogramming Non-Myelinating Cells into Myelin Gene-Expressing Cells
Source: PLoS One. 2010 Sep 27;5(9):e13023. doi: 10.1371/journal.pone.0013023 (PMC2946387; doi:10.1371/journal.pone.0013023)

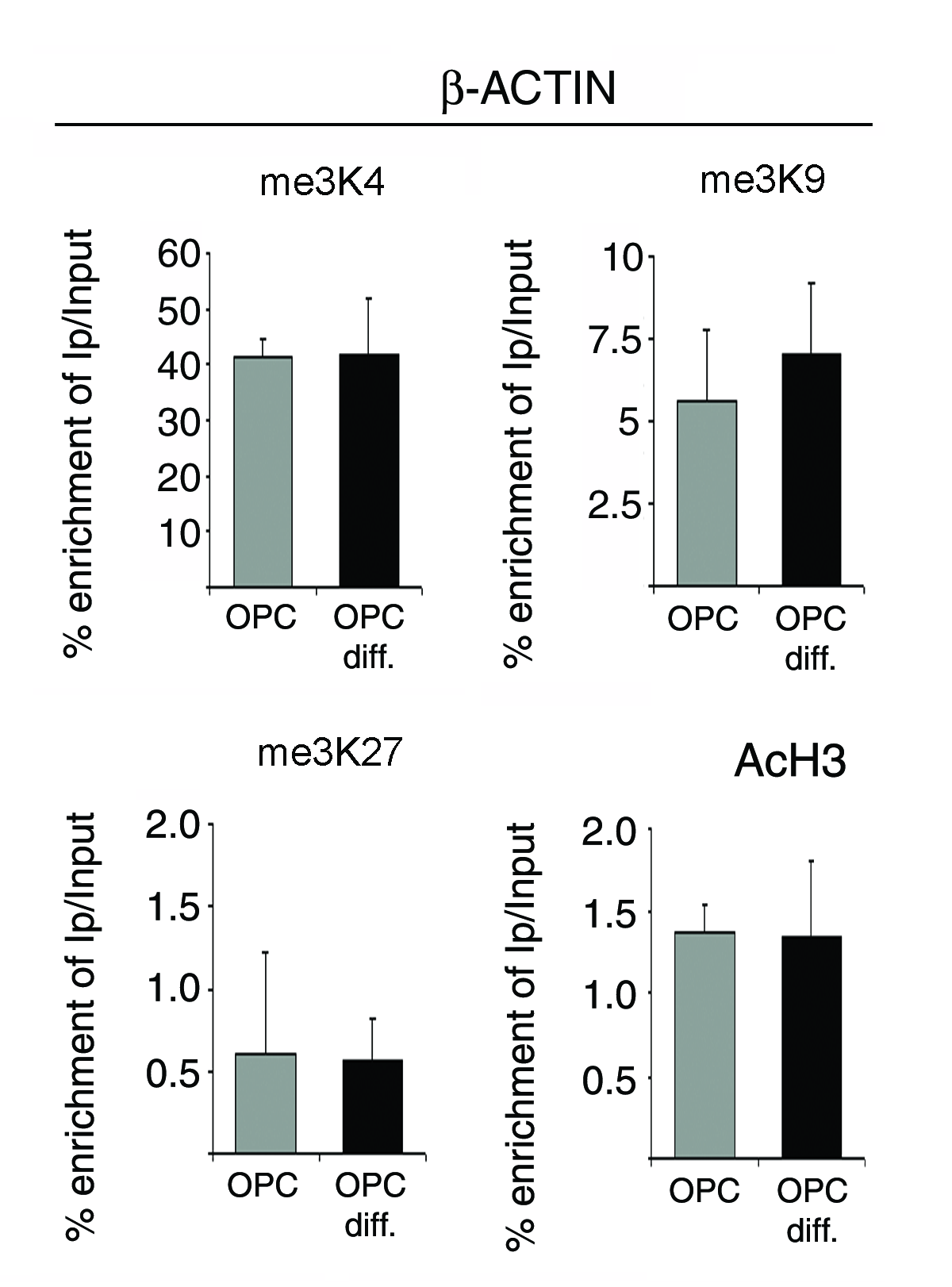

Supplement: Figure S1 — The pattern of histone code on the βactin gene was similar during oligodendrocyte progenitor differentiation. The pattern of histone modifications in beta-actin proximal promoters was analyzed in proliferating (OPC) and differentiating (OPC diff) rat primary oligodendrocyte cultures. Note that the histone code of this constitutively expressed gene was similar at the two stages of differentiation. (0.31 MB TIF) [file pone.0013023.s001.tif]
